# The Prisoner Who Cried Wolf, and Then Swallowed a Sprinkler Head

**DOI:** 10.5811/cpcem.2020.4.46449

**Published:** 2020-05-18

**Authors:** Matthew Hysell, Jennifer Finch, David E. McClendon

**Affiliations:** * Spectrum Health-Lakeland, Department of Emergency Medicine, St. Joseph, Michigan; † Holy Family Memorial Medical Center, Department of Emergency Medicine, Manitowoc, Wisconsin; ‡ Michigan State University College of Osteopathic Medicine, Department of Emergency Medicine, East Lansing, Michigan

**Keywords:** prisoner, swallow, incarceritis, malingering

## Abstract

**Case Presentation:**

A 37-year-old man presented from jail reporting foreign body ingestion of a sprinkler head. While initial radiography did not reveal the foreign body, subsequent imaging with computed tomography demonstrated the sprinkler head. When confronted with this discrepancy the patient admitted to having the sprinkler head in his possession and choosing to swallow it after his initial radiography.

**Discussion:**

This case demonstrates the importance of maintaining a high threshold for real illness in situations where there is suspected malingering, a situation not infrequently encountered in the emergency department.

## CASE PRESENTATION

A 37-year-old man presented to the emergency department (ED) from jail reporting foreign body ingestion. The patient reported other prisoners had repeatedly punched him; guards informed him a provider would see him the following day. He then reported swallowing his jail cell’s sprinkler head, successfully triggering evaluation. Physical exam demonstrated periorbital ecchymosis. Head computed tomography (CT) revealed facial fractures. Chest radiograph was unremarkable (Image A). The negative chest radiograph was discussed with the patient, who vehemently insisted he had swallowed the sprinkler head and reported globus. Chest CT demonstrated a metallic foreign body in the upper esophagus (Image B) at a level visualized by radiography. The patient later admitted to possession of the sprinkler head through his course in the ED, ultimately swallowing it covertly after the radiograph. Endoscopic removal was successful.

## DISCUSSION

Ingestion of foreign bodies by inmates and psychiatric patients is well documented.^1^,^2^ The delay in medical treatment following the patient’s assault offers a possible motive for his claim to have swallowed the foreign body. However, it was only after the patient arrived to the hospital and received medical care that he chose to swallow the sprinkler head. With negative initial testing it would have been easy for providers to have followed the actions of the villagers in “The Boy Who Cried Wolf” and to terminate further work-up. In this case the patient cried wolf so to speak, and then proceeded to release the wolf in the form of the sprinkler head ultimately demonstrated in the upper esophagus. While the patient’s rationale for the ingestion of the foreign body remains unclear, it is possible he wished to avoid return to jail where he had just been assaulted.^3^ This case demonstrates that maintaining a high threshold for real illness and listening to the patient, even in situations where malingering is suspected, is always necessary in the ED.

**CPC-EM Capsule**

What do we already know about this clinical entity?

The ingestion of foreign objects by inmates is well documented. Clinicians are also frequently faced with histories that may not be accurate.

What is the major impact of the image(s)?

First image demonstrates that initially provided history of foreign body ingestion was inaccurate. But upon subsequent imaging the foreign body was clearly visualized.

How might this improve emergency medicine practice?

*This case demonstrates the importance of maintaining a high threshold for real illness even in situations where malingering is suspected or even demonstrated*.

## Figures and Tables

**Image:**
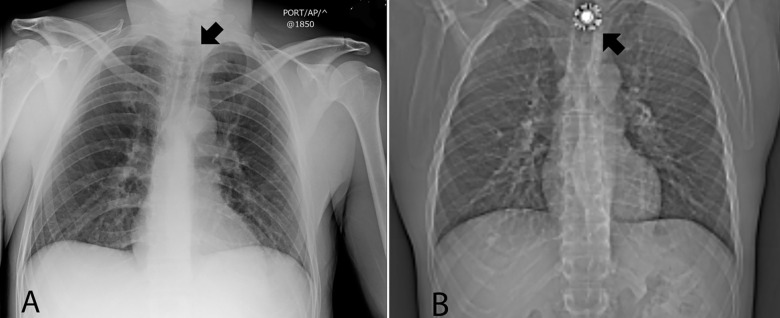
A. Normal initial chest radiograph absent of any ingested sprinkler head (arrow). B. Computed tomography scout view of chest now demonstrating the ingested sprinkler head (arrow).
